# A Fortnight's Festivities at Leicester

**Published:** 1913-01-04

**Authors:** 


### A FORTNIGHT'S FESTIVITIES AT LEICESTER.

### Opera and Cinema at the Royal Infirmary.

The Christmas festivities at this institution were ushered in at four o'clock on Christmas morning with a tour of the wards by the Nurses' Choral Society, when many of the old carols were sung. Every patient in the institution, adult and child, found on awaking a well-filled "Christmas stocking." In addition, every adult patient was supplied with an ounce of tobacco and given permission to smoke as his fancy pleased. Every ward was decorated. At 12.30 the usual Christmas dinner was served, while at four o'clock the members of the honorary staff and of the Board partook of tea in the "Odames" Ward, when Santa Claus, in the person of Dr. Harkness, the senior house surgeon, distributed presents from the electrically lighted ward Christmas tree. On Boxing Day Christmas fare was again served, and in the afternoon every patient was permitted to invite one guest for tea, provided from the Christmas Entertainment Fund.

On Friday, December 27, the nursing staff held a rehearsal of the operetta, referred to later on, in one of the wards, and on Saturday the patients were entertained in the Accident Ward with a musical programme arranged and organised by Mr. Percy H. Wykes. On Monday, the 30th, members of the nursing staff appeared in "The Sunshine Girl," in which the well-known Sunlight boxes played a prominent part, and subsequently presented "Between the Soup and the Savoury," by Gertrude Jennings, a comedy which was worthily rendered and heartily applauded. *Tableaux vivants* and songs concluded the entertainment. The publishers, Messrs. Samuel French, Ltd., kindly acquiesced in the comedy being performed without the usual guinea fee. On Tuesday, the 31st, the Grand Christmas tree and children's party was held in the large Out-patients' Hall. The tree, 20 feet in height, was kindly given by the authorities of the Desford Boys' Industrial School, and presented a charming appearance. Many friends of the institution supplied presents, which were supplemented by purchases from the Christmas Entertainment Fund. The Mayor and Mayoress gathered with Sir Edward Wood, and many friends of the institution, to distribute the gifts. On Wednesday the nurses held their customary dance, which was attended by many of the members of the honorary medical staff and other friends.

This, however, does not conclude the festivities, which will run well into January's second week, among the most interesting of which must be mentioned a cinematograph entertainment, kindly arranged, free of all cost, by Mr. R. T. Jupp, the managing director of the Provincial Cinematograph, Ltd. This mark of Mr. Jupp's interest arises from the fact that he was formerly one of the resident medical officers of the institution before he forsook the medical profession for a business career, which has not only been successful, but has been marked by a large-hearted sympathy with charitable enterprise. On Wednesday, January 8, Mrs. Forester, the wife of the much-respected Master of the Quorn pack, finally closes the festivities by giving her annual entertainment. All these arrangements have been supervised by Miss Vincent, the matron, who has won many eulogies for the organisation which she has built up. Mention must also be made of the unremitting services of Mr. Deacon, the clerk of works, under whose supervision the whole of the electrical arrangements for the various events have been carried out, and who has not only painted the necessary scenery for the nurses' operetta, but undertook the whole of the seating arrangements. Finally, be it noted, the funds for all these festivities have been provided by the friends of the institution, thanks to whom, and to our staff, all the patients will preserve the memory of their Christmas in the hospital to the end of their days.

